# New records of spirurid nematodes (Nematoda, Spirurida, Guyanemidae, Philometridae & Cystidicolidae) from marine fishes off New Caledonia, with redescriptions of two species and erection of *Ichthyofilaroides* n. gen.

**DOI:** 10.1051/parasite/2020003

**Published:** 2020-01-27

**Authors:** František Moravec, Jean-Lou Justine

**Affiliations:** 1 Institute of Parasitology, Biology Centre of the Czech Academy of Sciences Branišovská 31 370 05 České Budějovice Czech Republic; 2 Institut Systématique Évolution Biodiversité (ISYEB), Muséum National d’Histoire Naturelle, CNRS, Sorbonne Université, EPHE, Université des Antilles Rue Cuvier, CP 51 75005 Paris France

**Keywords:** Spirurida, Acanthuridae, Holocentridae, Hoplichthyidae, Serranidae, South Pacific

## Abstract

Recent examinations of spirurid nematodes (Spirurida) from deep-sea or coral reef marine fishes off New Caledonia, collected in the years 2006–2009, revealed the presence of the following five species: *Ichthyofilaroides novaecaledoniensis* (Moravec et Justine, 2009) n. gen., n. comb. (transferred from *Ichthyofilaria* Yamaguti, 1935) (females) (Guyanemidae) from the deep-sea fish *Hoplichthys citrinus* (Hoplichthyidae, Scorpaeniformes), *Philometra* sp. (male fourth-stage larva and mature female) (Philometridae) from *Epinephelus maculatus* (Serranidae, Perciformes), *Ascarophis* (*Dentiascarophis*) *adioryx* Machida, 1981 (female) (Cystidicolidae) from *Sargocentron spiniferum* (Holocentridae, Beryciformes), *Ascarophis* (*Ascarophis*) *nasonis* Machida, 1981 (males and females) from *Naso lituratus* and *N*. *unicornis* (Acanthuridae, Perciformes), and *Ascarophisnema tridentatum* Moravec et Justine, 2010 (female) from *Gymnocranius grandoculis* (Lethrinidae, Perciformes). Two species, *I*. *novaecaledoniensis* and *A*. *nasonis*, are redescribed based on light microscopical (LM) and scanning electron microscopical (SEM) examinations, the latter used in these species for the first time. Morphological data on the specimen of *A*. *tridentatum* from the new host species are provided. *Philometra* sp. (from *E*. *maculatus*) most probably represents a new gonad-infecting species of this genus. The newly established genus *Ichthyofilaroides* n. gen. is characterized mainly by the presence of a small buccal capsule and by the number and distribution of cephalic papillae in the female; it is the sixth genus in the Guyanemidae.

## Abstract (fr)

L’étude récente de nématodes Spirurida de poissons marins de mer profonde ou des récifs coralliens au large de la Nouvelle-Calédonie, collectés dans les années 2006-2009, a révélé la présence des cinq espèces suivantes : *Ichthyofilaroides novaecaledoniensis* (Moravec et Justine, 2009) n. gen., n. comb. (transféré depuis *Ichthyofilaria* Yamaguti, 1935) (femelles) (Guyanemidae) du poisson de profondeur *Hoplichthys citrinus* (Hoplichthyidae, Scorpaeniformes), *Philometra* sp. (larve mâle de quatrième stade et femelle mûre) (Philometridae) d’*Epinephelus maculatus* (Serranidae, Perciformes), *Ascarophis (Dentiascarophis) adioryx* Machida, 1981 (femelle) (Cystidicolidae) de *Sargocentron spiniferum* (Holocentridae, Beryciformes), *Ascarophis (Ascarophis) nasonis* Machida, 1981 (mâles et femelles) de *Naso lituratus* et *N. unicornis* (Acanthuridae, Perciformes), et *Ascarophisnema tridentatum* Moravec et Justine, 2010 (femelle) de *Gymnocranius grandoculis* (Lethrinidae, Perciformes). Deux espèces, *I. novaecaledoniensis* et *A. nasonis*, sont redécrites sur la base de la microscopie optique et de la microscopie électronique à balayage, cette dernière étant utilisée pour la première fois chez ces espèces. Des données morphologiques sur le spécimen d’*A. tridentatum* de la nouvelle espèce hôte sont fournies. *Philometra* sp. (d’*E. maculatus*) représente très probablement une nouvelle espèce infectant les gonades de ce genre. Le genre nouvellement établi *Ichthyofilaroides* n. gen. se caractérise principalement par la présence d’une petite capsule buccale et par le nombre et la répartition des papilles céphaliques chez la femelle. C’est le sixième genre des Guyanemidae.

## Introduction

Nematodes of the conventional order Spirurida Chitwood, 1933 [3, 8, 12] from marine fishes off New Caledonia have previously been dealt with in several papers [15–17, 28–43], mostly reporting various species of philometrids, camallanids and cystidicolids, less often gnathostomatids or a guyanemid. Nevertheless, taking into account the wealth of marine fish species in New Caledonian waters, it is expected that the nematode fauna of marine fishes, including spirurids, still remains poorly known.

Recent examinations of spirurid nematodes collected by J.-L. Justine and his students in marine fishes from off New Caledonia in the years 2006–2009 revealed the presence of five species of these parasites, two of which were previously insufficiently well described and two are newly recorded in New Caledonian waters. Results of this study are presented herein.

## Materials and methods

Fish were caught off New Caledonia by various means; fish from the deep-sea campaign were frozen-thawed. We generally used the “wash” method [18]. The nematodes for morphological studies were fixed in hot 4% formalin or 70% ethanol. For light microscopical examination (LM), they were cleared with glycerine. Drawings were made with the aid of a ZEISS microscope drawing attachment. Specimens used for scanning electron microscopical (SEM) examination were postfixed in 1% osmium tetroxide (in phosphate buffer), dehydrated through a graded acetone series, critical-point-dried and sputter-coated with gold; they were examined using a JEOL JSM-7401F scanning electron microscope at an accelerating voltage of 4 kV (GB low mode). All measurements are in micrometres unless otherwise indicated. The fish nomenclature adopted follows FishBase [11].

## Results

Family Guyanemidae Petter, 1974

### *Ichthyofilaroides* n. gen.

urn:lsid:zoobank.org:act:6E8F1B66-B534-4320-805F-5DCD273A8FFF

*Diagnosis*: Dracunculoidea, Guyanemidae, Travassosneminae. Body of female cylindrical, with narrowed anterior and posterior ends; cephalic end rounded, posterior end pointed. Cuticle smooth. Oral aperture circular, surrounded by four small submedian cephalic papillae arranged in two circles and by pair of lateral amphids. Small but distinct, simple buccal capsule present. Oesophagus divided into anterior muscular and short posterior glandular portions; latter with long posteriorly oriented appendix. Intestine ending posteriorly in ligament attached to body wall. Amphidelphic. Posterior ovary much larger than anterior one, extending almost to anterior end of tail. Uterus continuous, filled with larvae. Vulva functional, situated in posterior half of body. Male not known. Parasites of musculature, surface of visceral organs and body cavity of marine fishes.

Type species: *I*. *novaecaledoniensis* (Moravec et Justine, 2009) n. comb.

### *Ichthyofilaroides novaecaledoniensis* (Moravec et Justine, 2009) n. comb. Figures 1, 2

Host: Lemon ghost flathead *Hoplichthys citrinus* Gilbert (Hoplichthyidae, Scorpaeniformes).

**Figure 1:**
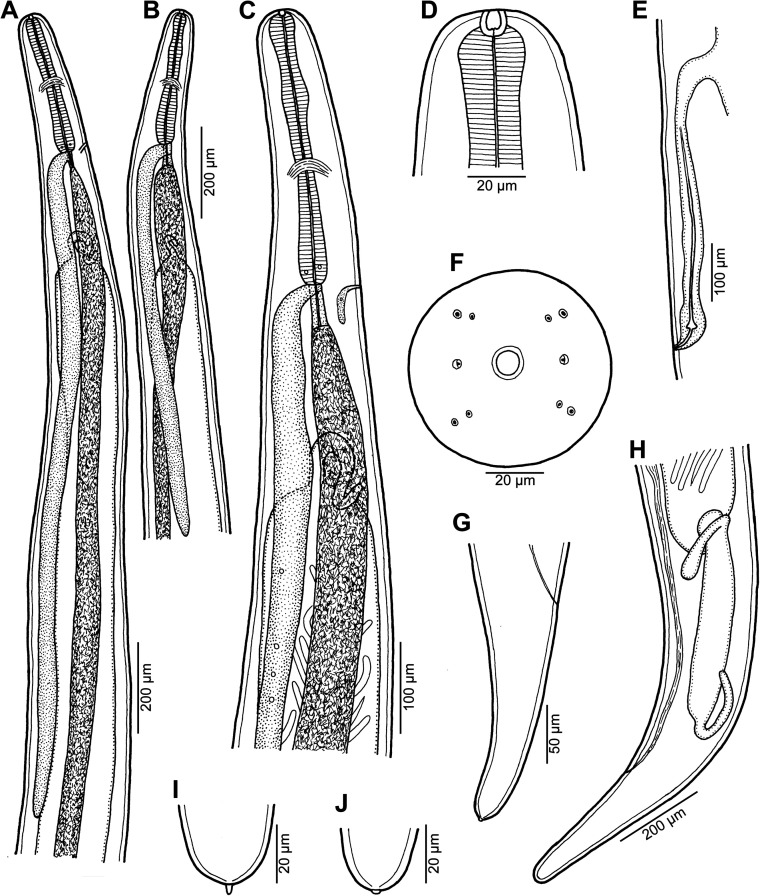
*Ichthyofilaroides novaecaledoniensis* (Moravec et Justine, 2009) n. comb., gravid female from *Hoplichthys citrinus*. (A, B) Anterior ends of two different specimens, lateral views; (C) anterior end, lateral view (enlarged); (D) cephalic end, lateral view; (E) vulva and vagina, lateral view; (F) cephalic end, apical view; (G) tail, lateral view; (H) posterior end, lateral view; (I, J) tail tips of two different specimens.

**Figure 2:**
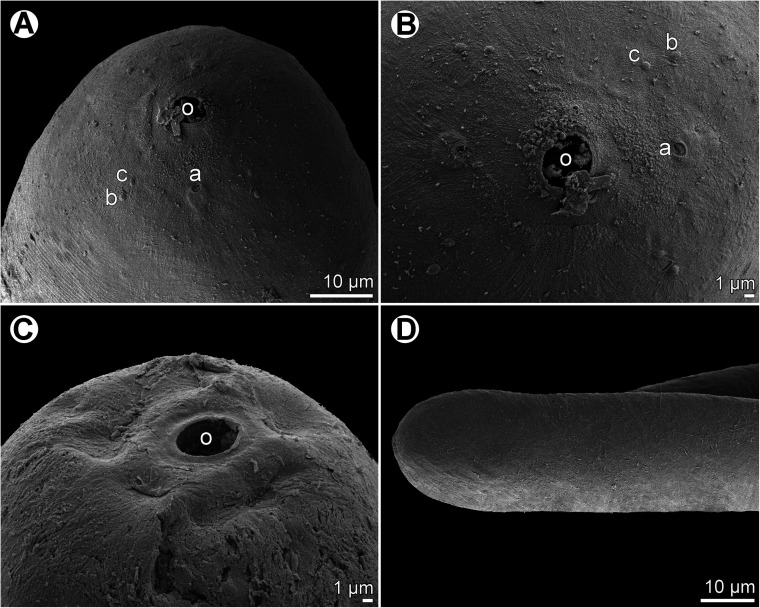
*Ichthyofilaroides novaecaledoniensis* (Moravec et Justine, 2009) n. comb., scanning electron micrographs of gravid female. (A) Cephalic end, subapical view; (B) region of oral aperture, apical view; (C) cephalic end (another specimen), subapical view; (D) tail end of specimen without terminal caudal projection. (a) Amphid; (b) submedian cephalic papilla of outer circle; (c) submedian cephalic papilla of inner circle; (o) oral aperture.

Site of infection: Unknown with precision, parasites obtained from wash of organs in abdominal cavity of frozen-thawed fish.

Locality: Off New Caledonia, deep-sea cruise TERRASSE [54]. JNC2776, station CP3048, 23°44′S, 168°16′E, 19 October 2008, depth 380–400 m; JNC2791, JNC2795, station CP3089, 22°17′S, 167°12′E, 25 October 2008, depth 390–410 m.

Prevalence, intensity and details of fish: 14% (3 fish infected/22 fish examined); 1–2 nematodes. The infected fish (JNC2776, JNC2791 and JNC2795) were 210–215 mm in total length, 36–38 g in weight.

Deposition of voucher specimens: Muséum National d’Histoire Naturelle, Paris, France (1 specimen and 2 body fragments in 70% ethanol, MNHN JNC2776, JNC2791 and JNC2795).

#### Description

*Female* (four gravid specimens): Body filiform, whitish, 20.21–26.33 mm long, maximum width near middle of body 245–354, tapering at both ends. Cuticle smooth. Anterior end bluntly rounded (Figs. 1A–1C and 2A). Cephalic papillae small, indistinct in lateral view. Oral aperture small, circular, surrounded by cephalic papillae arranged in two circles: four larger submedian papillae of outer circle and four smaller submedian papillae of inner circle; pair of small lateral amphids present (Figs. 1F and 2A–2C). Small buccal capsule 6 long and 7–9 wide present (Fig. 1D). Muscular oesophagus narrow, almost cylindrical, 261–339 long and 24–39 wide at its posterior portion, sometimes forming small bulbous inflation at its anterior end; inflation 30–33 long and 24–36 wide. Length of muscular oesophagus represents 1.1%–1.4% of body length. Glandular oesophagus very short, 27–60 long and 18–45 wide, provided with free posterior appendix 1.02–1.64 mm long and 45–48 in maximum width (Figs. 1A–1C). Nerve ring encircling muscular oesophagus 171–219 from anterior extremity; excretory pore at level of junction of muscular and glandular portions of oesophagus (Figs. 1A–1C); deirids not observed. Intestine light-coloured, 4.62–5.71 mm long, ending blindly, continuing as narrow ligament attached ventrally to body wall at distance of 272–300 from posterior extremity. Amphidelphic. Vulva non-elevated or slightly elevated, located 14.89–18.75 mm from anterior end of body, at 65%–74% of body length (Fig. 1E). Vagina tubular, 213–843 long and 21–24 wide, directed anteriorly from vulva; small oval ovijector present, 30–75 long and 18–48 wide (Fig. 1E). Uterus occupying majority of space of body, being filled with many eggs, developing embryos and free larvae; larvae about 150 long and 9–12 wide. Anterior ovary small, narrow; posterior ovary fairly broad, forming coils (Figs. 1A–1C). Caudal end conical, with or without minute terminal cuticular projection (Figs. 1G–1J and 2D).

#### Remarks

Moravec and Justine [33] described the dracunculoid *Ichthyofilaria novaecaledoniensis* Moravec et Justine, 2009 from a single subgravid female obtained from the flesh near gills of *H*. *citrinus* off New Caledonia, which was studied solely by LM. Since the available specimen was fairly damaged, some of its important morphological features were given inaccurately, as visible from the present study, or were not observed at all. The four newly collected specimens (all gravid females) of this species made it possible to redescribe it based on both LM and SEM examinations.

In contrast to the original description, the present study has revealed the presence of a small, but distinct buccal capsule in this species, which indicates a different generic appurtenance of this nematode. In addition, the shape of the oral aperture, the number and arrangement of cephalic papillae and the presence of amphids and the functional vulva in this nematode are reported for the first time. The present study has also shown that the posterior appendix of the glandular oesophagus is much longer as compared with the original description.

*Ichthyofilaria* Yamaguti, 1935 was established by Yamaguti [61] to accommodate *I*. *dasycotti* Yamaguti, 1935, a species described solely from females from the abdominal cavity of *Dasycottus setiger* Bean (Psychrolutidae, Scorpaeniformes) in the Sea of Japan. The genus was placed in the Philometridae [8, 14, 61, 62], but later it was transferred to the family Guyanemidae Petter, 1974, subfamily Travassosneminae Moravec, 2006 [26]. To date, *Ichthyofilaria* includes six, mostly little-known species reported from the abdominal cavity and different tissues of marine fishes: *I*. *argentinensis* Incorvaia, 1999 from gadiform fishes in the western South Atlantic Ocean [7, 13, 58]; *I*. *bergensis* (Wülker, 1930) Køie, 1993 from gadiform fishes in the eastern North Atlantic Ocean and the Mediterranean Sea [7, 20, 59]; *I*. *canadensis* Appy, Anderson et Khan, 1985 from perciform fishes in the western North Atlantic Ocean [4]; *I*. *dasycotti* (type species; see above); *I*. *japonica* Moravec et Nagasawa, 1985 from a scorpaeniform fish off Japan [47]; and *I*. *novaecaledoniensis* from the scorpaeniform fish *Hoplichthys citrinus* off New Caledonia [33].

However, as revealed in this study, *I*. *novaecaledoniensis* differs substantially from other congeners in possessing a small buccal capsule, by which it shows affinities to species of the dracunculoid family Skrjabillanidae Shigin et Shigina, 1958, parasites of freshwater fishes [25]. It should be noted that skrjabillanids are similar to species of *Ichthyofilaria* also in having the glandular oesophagus provided with a posterior appendix [25] and the males of both these nematode groups possess a copulatory plate instead of spicules [7, 23, 25, 55, 56]. On the other hand, in contrast to *I*. *novaecaledoniensis* and other congeners, the skrjabillanid females are monodelphic (vs. didelphic), possessing the vulva situated in the oesophageal region (vs. in the posterior half of body) and the female tail tip of most species bears three projections. Moreover, all skrjabillanids are parasites of freshwater fishes (see above). Consequently, *I*. *novaecaledoniensis* is not a species belonging to the Skrjabillanidae.

Besides the presence of a buccal capsule (see above), the female of *I*. *novaecaledoniensis* also differs from congeners in the number and arrangement of cephalic papillae. According to Timi et al. [58], there are eight papillae in the outer circle arranged in four submedian pairs and two (but probably four [26]) submedian single papillae in the inner circle in *I*. *argentinensis* females (vs. four submedian papillae in the outer circle and four submedian papillae in the inner circle in *I. novaecaledoniensis*). Four elevated submedian pairs of papillae were reported for females of *I*. *japonica* [47], whereas four poorly defined submedian papillae were found in females of *I*. *bergensis* and *I*. *canadensis* [4, 20]; the cephalic papillae of *I*. *dasycotti* have not yet been studied.

Considering other dracunculoids, based on the number and arrangement of cephalic papillae in the female, *I*. *novaecaledoniensis* resembles only *Lucionema balatonense* Moravec, Molnár et Székely, 1998 (Lucionematidae), a parasite of the swimbladder wall of the freshwater percid *Sander lucioperca* (Linnaeus) in Europe and *Lockenloia sanguinis* Adamson et Caira, 1991 (*genus incertae sedis*), an inadequately described parasite from the heart of the nurse shark *Ginglymostoma cirratum* (Bonnaterre) in the western North Atlantic Ocean [1, 46]; however, both of these species differ from *I*. *novaecaledoniensis* in the structure of the oesophagus and for *L*. *balatonense* also in the absence of the buccal capsule and in that its female genital tract is monodelphic (vs. didelphic).

Therefore, we consider it necessary to erect a new genus, *Ichthyofilaroides* n. gen., to accommodate *I*. *novaecaledoniensis*. This new genus is differentiated from *Ichthyofilaria* mainly by the presence of the buccal capsule and the number and arrangement of the cephalic papillae. Since the buccal capsule has not yet been described in any species of the Guyanemidae, it may be necessary to create a new family for *Ichthyofilaroides* n. gen. in the future. Nevertheless, since the male of its type species remains unknown, we provisionally assign *Ichthyofilaroides* to the family Guyanemidae, subfamily Travassosneminae.

In addition to *Ichthyofilaroides* n. gen., species of the following six genera are placed in the family Guyanemidae [26]: *Guyanema* Petter, 1974, *Histodytes* Aragort, Álvarez, Iglesias, Leiro et Sanmartín, 2002, *Moravecia* Ribu et Lester, 2004, *Pseudodelphis* Adamson et Roth, 1990 (syn. *Syngnathinema* Moravec et al., 2001 [48]), *Ichthyofilaria* and *Travassosnema* Costa, Moreira et Oliveira, 1991 [2, 5, 9, 52, 53, 61].

It should be noted that, even though the molecular phylogeny of some fish dracunculoids has been studied (e.g. [10, 23, 50, 57, 60]), no such studies have so far been performed on representatives of the Guyanemidae.

Family Philometridae Baylis et Daubney, 1926

### *Philometra* sp. Figure 3

Host: Highfin grouper *Epinephelus maculatus* (Bloch) (Serranidae, Perciformes).

**Figure 3:**
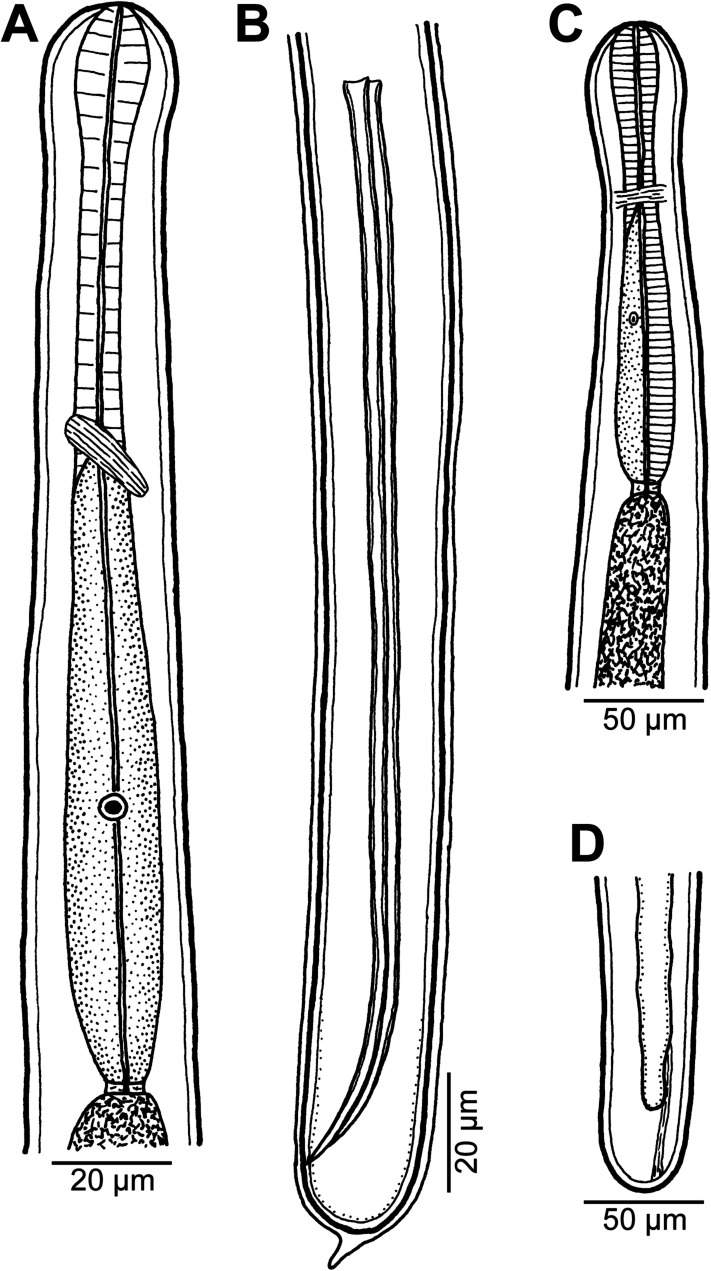
*Philometra* sp. from *Epinephelus maculatus*. (A, B) Anterior and posterior ends of male fourth-stage larva, respectively, lateral views; (C, D) anterior and posterior ends of mature female, respectively, lateral views.

Site of infection: Unknown with precision, parasites obtained from wash of organs in abdominal cavity.

Locality: Near Récif Toombo, off New Caledonia, 22°34′S, 166°28′E.

Prevalence, intensity and details about fish: 58% (2 fish infected/32 fish examined); 1 specimen per fish. Fish JNC3031, total length 387 mm, weight 716 g, 1 September 2009; JNC3066, total length 449 mm, weight 959 g, 30 September 2009.

Deposition of voucher specimens: Muséum National d’Histoire Naturelle, Paris, France (two specimens, MNHN JNC3031 and JNC3066).

#### Description

*Male fourth-stage larva* (one specimen): Body of specimen undergoing last moult 3.17 mm long, maximum width 30, slightly narrowed posterior to cephalic end (Fig. 3A). Width of cephalic end 24, of narrowed cephalic region 21 and that of caudal end 21. Entire oesophagus 186 long, slightly inflated at anterior end, 15 in maximum width. Nerve ring and cell nucleus of oesophageal gland 57 and 132, respectively, from anterior extremity. Spicules equal, 189 long, representing 6% of body length (Fig. 3B). Gubernaculum not yet developed. Newly formed caudal end broadly oval, still inside moulted cone-shaped larval cuticle (Fig. 3B).

*Mature female* (one specimen): Body whitish, 3.17 mm long, maximum width 81, slightly narrowed posterior to rounded cephalic end (Fig. 3C). Width of cephalic end 39, of narrowed cephalic region 36. Entire oesophagus 216 long, representing 7% of body length, its maximum width 24. Nerve ring and cell nucleus of oesophageal gland 75 and 150, respectively, from anterior extremity (Fig. 3C). Uterus poorly developed, empty. Vulva present, being situated 2.26 mm from anterior extremity, at 71% of body length; short vagina directed anteriorly from vulva. Caudal end rounded, 30 wide (Fig. 3D).

#### Remarks

To date, 14 nominal species of *Philometra* Costa, 1845 have been reported from marine fishes in New Caledonia [28, 32, 33, 37–39], of which *P*. *cephalopholidis* Moravec et Justine, 2015, *P*. *cyanopodi* Moravec et Justine, 2008, *P*. *fasciati* Moravec et Justine, 2008, *P*. *ocularis* Moravec, Ogawa, Suzuki, Miyazaki et Donai, 2002 and *P*. *piscaria* Moravec et Justine, 2014 were recorded from fishes of the family Serranidae. Of the species with described males, only *P*. *cyanopodi* from the ovary of *Epinephelus cyanopodus* (Richardson) has a length of spicules (183–229 μm) similar to that of the present male larva from *E*. *maculatus* (189 μm) and the body lengths are also similar (2.72–3.59 mm vs. 3.17 mm). However, since other taxonomically important features (especially the structure of the gubernaculum) of the present specimen are not known, and considering the high degree of host specificity of *Philometra* species parasitizing serranid fishes [27, 45], the present nematode is unidentifiable to species. Nevertheless, the finding of *Philometra* sp. in *E*. *maculatus* represents the first record of a philometrid in this host species.

Family Cystidicolidae Skryabin, 1946

### *Ascarophis* (*Dentiascarophis*) *adioryx* Machida, 1981

Syn.: *Ascarophis holocentri* Parukhin, 1984.

Host: Sabre squirrelfish *Sargocentron spiniferum* (Forsskål) (Holocentridae, Beryciformes).

Site of infection: Digestive tract.

Locality: Récif Crouy, off New Caledonia (collected 13 May 2008).

Prevalence, intensity and details of fish: 1 fish infected/4 fish examined; 1 nematode. The infected fish (JNC2530) was 185 mm in total length and 185 g in weight.

Deposition of voucher specimen: Muséum National d’Histoire Naturelle, Paris, France Paris (1 specimen, MNHN JNC2530).

#### Remarks

*Ascarophis adioryx* has already been reported from two species of holocentrid fish hosts off New Caledonia, *Sargocentron spiniferum* and *Neoniphon sammara* (Forsskål) and, on the basis of available specimens, the species was redescribed in detail based on LM and SEM [34]. The present material contained only a single specimen (gravid female); therefore, we have refrained from describing it.

Originally this nematode species was described from *S*. *spiniferum* and *S*. *rubrum* (Forsskål) in the Philippine Sea off the Palau Islands (Republic of Palau), Oceania [21] and later it was recorded under the synonym *Ascarophis holocentri* Parukhin, 1984 from *S*. *spiniferum* and *S*. *punctatissimum* (Cuvier) in the Red Sea [51]. Consequently, *A*. *adioryx* seems to be a specific parasite of holocentrid fishes in the Indo-Pacific region.

### *Ascarophis* (*Ascarophis*) *nasonis* Machida, 1981 Figures 4–6

Hosts: Orangespine unicornfish *Naso lituratus* (Forster) and bluespine unicornfish *Naso unicornis* (Forsskål) (Acanthuridae, Perciformes).

**Figure 4:**
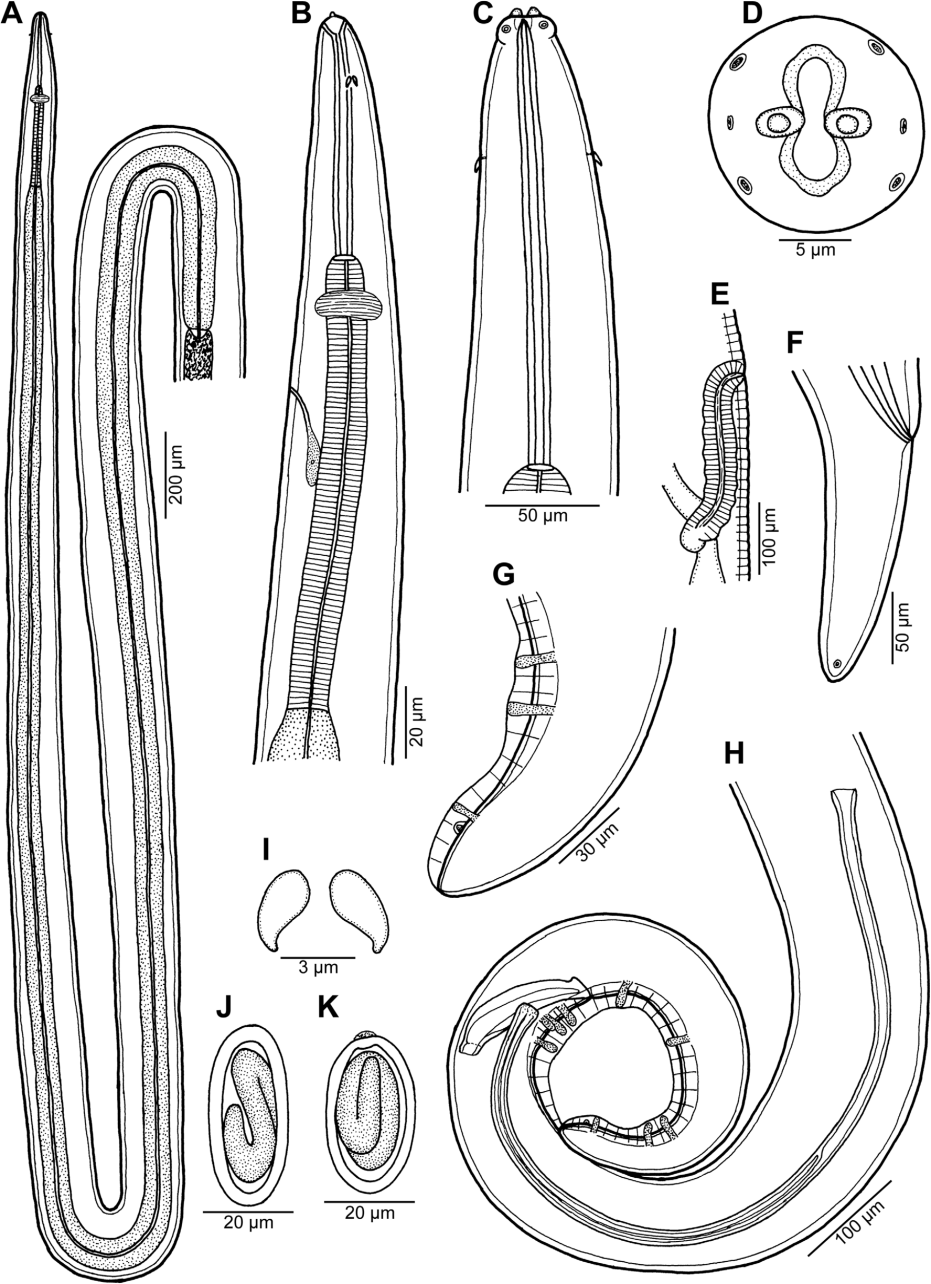
*Ascarophis* (*Ascarophis*) *nasonis* Machida, 1981 from *Naso* spp. (A) Oesophageal part of female body, dorsoventral view; (B) anterior end of female, lateral view; (C) anterior end of female (another specimen, enlarged), dorsoventral view; (D) cephalic end, apical view; (E) vulva and vagina, lateral view; (F) tail of female, lateral view; (G) posterior end of male tail, lateral view; (H) posterior end of male, lateral view; (I) deirid; (J, K) egg. (A) and (C)–(J) from *N*. *lituratus*; (B) and (K) from *N*. *unicornis*.

Site of infection: Anterior part of intestine (*N*. *lituratus*) and digestive tract (*N*. *unicornis*).

Localities: Near Ilôt Signal (collected 30 May 2006) (*N*. *lituratus*) and near Ilôt Goëland (collected 26 October 2007) (*N*. *unicornis*), both off New Caledonia.

Prevalence, intensity and details of fish: *N*. *lituratus*: 1 fish infected/2 fish examined; 5 nematodes. *N*. *unicornis*: 5% (1/19); 5. *N*. *lituratus*, fish JNC1843, total length 275 mm, weight 590 g; *N*. *unicornis*, fish JNC2345, total length 435 mm, weight 1800 g.

Deposition of voucher specimens: Muséum National d’Histoire Naturelle, Paris, France (6 specimens, MNHN JNC1843, JNC2345) and Helminthological Collection, Institute of Parasitology, Biology Centre of the Czech Academy of Sciences, České Budějovice, Czech Republic (1 specimen, IPCAS N–1206).

#### Description

**Figure 5:**
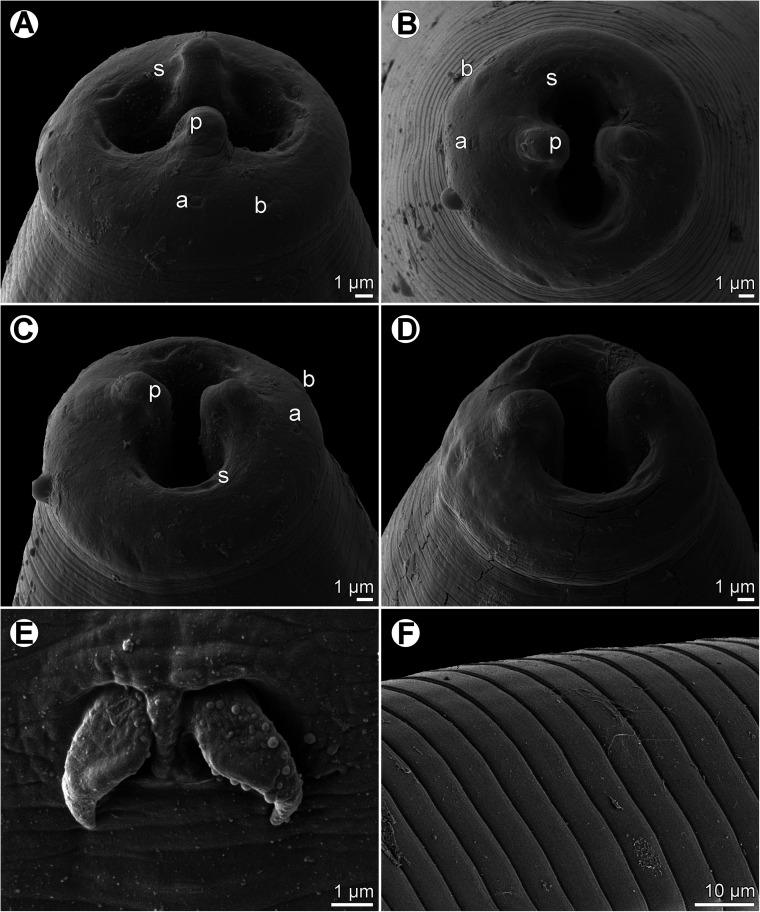
*Ascarophis* (*Ascarophis*) *nasonis* Machida, 1981 from *Naso* spp., scanning electron micrographs. (A, B) Cephalic end, lateral and apical views, respectively; (C, D) cephalic end, dorsoventral views (two different specimens); (E) deirid; (F) detail of cuticle at middle part of body. (a) Amphid; (b) cephalic papilla; (p) pseudolabium; (s) sublabium.

**Figure 6:**
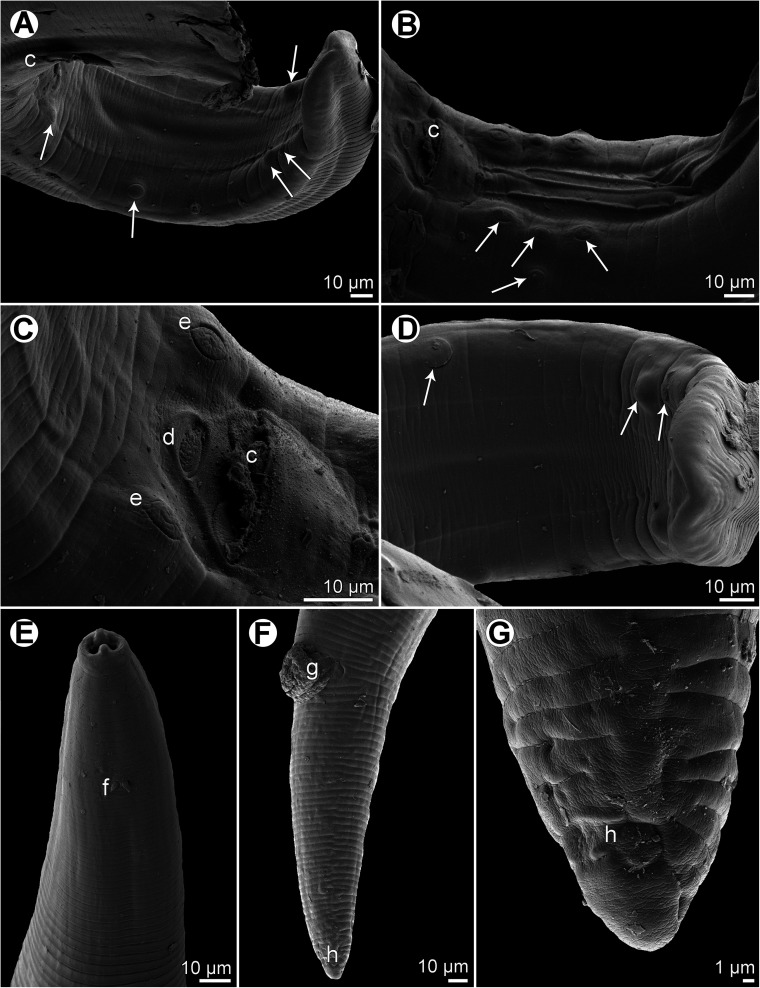
*Ascarophis* (*Ascarophis*) *nasonis* Machida, 1981 from *Naso* spp., scanning electron micrographs. (A) Tail of male, sublateral view (arrows indicate postanal papillae); (B) precloacal region of male, ventrolateral view (arrows indicate preanal papillae); (C) region of cloaca, subventral view; (D) posterior part of male tail, ventral view (arrows indicate postanal papillae); (E) anterior end of body, lateral view; (F) tail of female, lateral view; (G) posterior end of female tail, lateral view. (c) Cloacal aperture; (d) median depression on posterior cloacal lip; (e) papillae of first postanal pair; (f) deirid; (g) anus; (h) phasmid.

*General*: Small, whitish nematodes. Maximum width of body near its middle. Cuticle thick, with distinct transverse striations (Figs. 5F, 6E, 6F). Cephalic end rounded, with two pseudolabia with terminal protrusions possessing rounded tips (Figs. 4B, 4C, 5A–5D and 6E). Oral aperture oval, dorsoventrally elongate, slightly narrowed equatorially (Figs. 4D, 5B). Lateral pseudolabia rather large, each provided with rounded terminal protrusion; in apical view, inner parts of pseudolabia partly cover mouth (Figs. 4D, 5B). Oral aperture surrounded by continuous mound bearing four submedian cephalic papillae and pair of lateral amphids; submedian sublabia poorly developed, narrow, appearing to be fused together dorsally and ventrally (Figs. 4D, 5A–5D). Vestibule (stoma) long, almost cylindrical, with well-developed funnel-shaped anterior prostom visible in lateral view (Fig. 4B). Glandular oesophagus conspicuously long, 6–24 times longer than muscular; both parts of oesophagus distinctly separated from each other (Figs. 4A, 4B). Nerve ring encircles muscular oesophagus near its anterior end; excretory pore located somewhat posterior to level of nerve ring; deirids small, bifurcate, situated approximately at 1/3 of vestibule length; only 2 posteriorly oriented arms of each deirid visible on surface of cuticle (Figs. 4B, 4C, 4I and 5E).

*Male* (two specimens from *N*. *lituratus*. Measurements of one specimen from *N*. *unicornis* in parentheses): Length of body 14.42–14.43 (15.61) mm, maximum width 150–163 (204). Height of pseudolabia 3 (3). Vestibule including prostom 165–189 (171) long; prostom 15 (–) long and 12 (–) wide. Length of muscular oesophagus 285–291 (282), maximum width 24–27 (27); length of glandular oesophagus 5.51–6.05 (5.59) mm, maximum width 60–66 (72); length ratio of muscular and glandular parts of oesophagus 1:19–21 (1:20). Length of entire oesophagus and vestibule represents 41%–45% (39%) of total body length. Deirids, nerve ring and excretory pore 60–66 (42), 207–225 (204) and 372–375 (–), respectively, from anterior extremity. Posterior end of body spirally coiled, provided with narrow caudal alae (Fig. 4H). Preanal papillae: four pairs of subventral pedunculate papillae present, being arranged in two longitudinal rows (more subventral row formed by three pairs and one pair more lateral, at same level as third more subventral pair) (Figs. 4H and 6B). Postanal papillae: six pairs present, including five pairs of pedunculate subventral papillae and one pair of minute ventral sessile papillae located approximately at level of last pair of subventrals (Figs. 4G, 4H, 6A, 6C and 6D). Ventral cuticular ridges (area rugosa) anterior to cloaca well developed, consisting of about four longitudinal lines of elevated cuticle (Fig. 6B). Large (left) spicule 846–870 (858) long, with obtuse distal tip; small (right) spicule boat-shaped, 144–147 (141) long, with small dorsal barb on distal end rounded distal end (Fig. 4H). Length ratio of spicules 1:5.8–6.0 (1:6.1). Tail conical, 354 (394) long, with rounded tip (Figs. 6A and 6D).

*Female* (3 ovigerous specimens from *N*. *lituratus*. Measurements of four ovigerous specimens from *N*. *minor* in parentheses): Length of body 16.91–19.57 (8.51–11.26) mm, maximum width 177–204 (109–190). Height of pseudolabia 3 (3). Vestibule including prostom 180–183 (126–171) long; prostom 15 (12) long and 12 (12) wide. Length of muscular oesophagus 264–288 (255–330), maximum width 17–30 (21–24); length of glandular oesophagus 5.86–6.94 (1.50–2.03) mm, maximum width 75 (39–75); length ratio of muscular and glandular parts of oesophagus 1:22–24 (1:6). Length of entire oesophagus and vestibule represents 37–38 (22–24)% of total body length. Deirids, nerve ring and excretory pore 45–63 (39–48), 210–216 (126–189) and 315–321 (246–429), respectively, from anterior extremity. Tail conical, 195–228 (150–225) long, with rounded tip without any terminal appendage (Figs. 4F, 6F, 6G). Vulva mostly postequatorial, situated 10.17–14.42 (4.28–5.96) mm from anterior end of body, at 60%–74% (49%–53%) of body length; vulval lips not elevated (Fig. 4E). Vagina directed posteriorly from vulva (Fig. 4E). Amphidelphic. Anterior ovary and uterus not extending anteriorly to oesophageal part of body. Uterus filled with many eggs. Mature eggs (containing larvae) elongate-oval, thick-walled, size 42–45 × 18–21 (33–39 × 18–24); thickness of egg wall 3 (3); surface of eggs without filaments (Figs. 4J and 4K).

#### Remarks

The present specimens are morphologically similar to *A*. *nasonis* Machida, 1981, described from the same two hosts species, *N*. *unicornis* (now considered to be probably a synonym of *N*. *minor* [11]) (type host) and *N*. *lituratus*, from off southern Japan (Okinawa Prefecture) and Palau Islands [21] and there is no doubt that they belong to this species. Based on LM, Machida [21] gave a relatively good description of this species, but he could not observe some morphological details visible only with the use of SEM. The present SEM examination of this species revealed, for the first time, the presence of ventral precloacal cuticular ridges in the male, the presence and location of phasmids in the female and showed the exact structure of the nematode’s cephalic end.

This is the second finding of *A*. *nasonis* since its original description [21] and the first record of this nematode from fishes in New Caledonian waters. Previously two other congeneric species were reported from New Caledonia, *Ascarophis adioryx* from holocentrids (see above) and *A*. *richeri* Moravec et Justine, 2007 from the scorpaeniform fish *Hoplichthys citrinus* [30]. Whereas *A*. *nasonis* can be easily distinguished from the otherwise similar *A*. *adioryx* by the longer left spicule (810–1100 μm vs. 516–610 μm) and the mouth structure (mouth without dorsal and ventral median projections and pseudolabia without conspicuous inner dorsoventral extensions), both *A*. *adioryx* and *A*. *nasonis* differ from *A*. *richeri* mainly in possessing nonfilamented eggs (vs. eggs with long filaments on either pole) and some other features such as the mouth structure, shape of deirids and the structure of ventral precloacal ridges in the male.

Based on the structure of the mouth, each deirid with two posteriorly oriented arms separated one from another by the body cuticle, an unusually long glandular oesophagus, a fairly long female tail, the obtuse distal tip of the left spicule and the shape of eggs, *A*. *nasonis* is similar to the recently described *A*. *scatophagi* Moravec, Yooyen et Sanprick, 2018, a parasite of *Scatophagus argus* (Linnaeus) (Scatophagidae, Perciformes) in the Gulf of Thailand [49]. However, in contrast to *A*. *nasonis*, *A*. *scatophagi* possesses a distinctly shorter left spicule (555–642 μm vs. 846–1100 μm) and more numerous (about 10 vs. about 4) longitudinal precloacal cuticular ridges; moreover, the hosts of these two nematode species belong to different fish families (Acanthuridae vs. Scatophagidae).

In contrast to the majority of congeneric species, the fully developed (larvated) eggs of *A*. *nasonis* were described and illustrated to be conspicuously elongate [21]; similar, markedly elongate-oval eggs were also described for *A*. *longiovata* Moravec et Klimpel, 2009 and *A*. *scatophagi* [44, 49]. Nevertheless, although the fully developed eggs of the present specimens of *A*. *nasonis* from *N*. *lituratus* are typical in shape of this species (i.e. conspicuously elongate) (Fig. 4J), those of *A*. *nasonis* from *N*. *unicornis* are relatively wider with respect to their length and some of them appear to bear a small, poorly developed swelling on one pole (Fig. 4K). Since the gravid *A*. *nasonis* females from *N*. *unicornis* were smaller and less developed (with smaller numbers of eggs in uteri) as compared with those from *N*. *lituratus*, it may well be that the egg shape is influenced by the state of the female development.

*Ascarophis nasonis* has not been recorded previously from New Caledonian waters.

### *Ascarophisnema tridentatum* Moravec et Justine, 2010 Figures 7–9

Host: Blue-lined large-eye bream *Gymnocranius grandoculis* (Valenciennes) (Lethrinidae, Perciformes).

**Figure 7:**
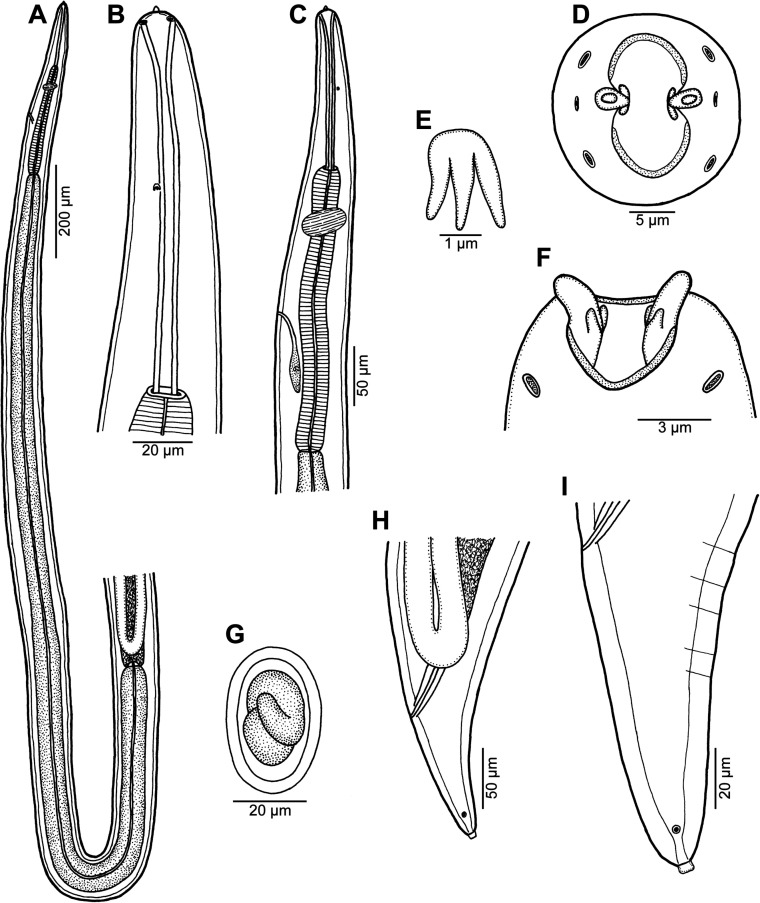
*Ascarophisnema tridentatum* Moravec et Justine, 2010 from *Gymnocranius grandoculis*, female.

Site of infection: Stomach.

Locality: Near Récif Toombo, off Nouméa, New Caledonia (collected 15 May 2007).

Prevalence, intensity and details of fish: 1 fish infected/11 fish examined; 1 nematode. The infected fish, JNC2166, was 595 mm in total length and 3800 g in weight.

Deposition of voucher specimen: Muséum National d’Histoire Naturelle, Paris, France (body fragment of 1 specimen, MNHN JNC2166).

#### Description

**Figure 8:**
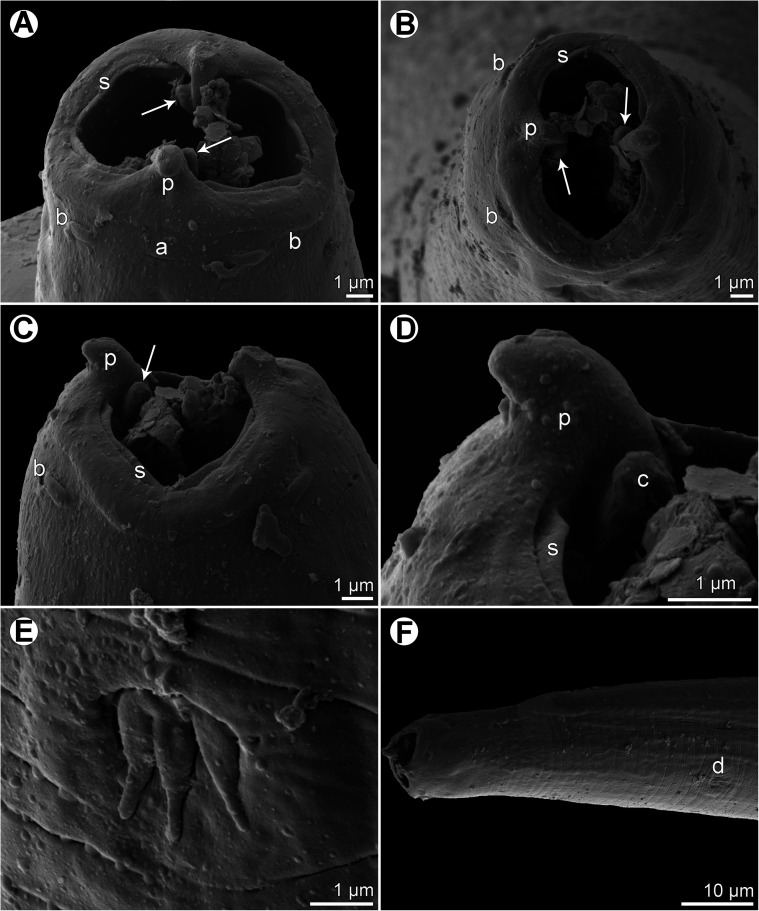
*Ascarophisnema tridentatum* Moravec et Justine, 2010 from *Gymnocephalus grandoculis*, scanning electron micrographs of female. (A, B) Cephalic end, lateral and apical views, respectively (arrows indicate tooth-like pseudolabial extensions); (C) cephalic end, dorsoventral view (arrow indicates tooth-like pseudolabial extension); (D) enlarged region of pseudolabium, dorsoventral view; (E) deirid; (F) anterior end of body, sublateral view. (a) Amphid; (b) cephalic papilla; (d) deirid; (p) pseudolabium; (s) sublabium.

**Figure 9:**
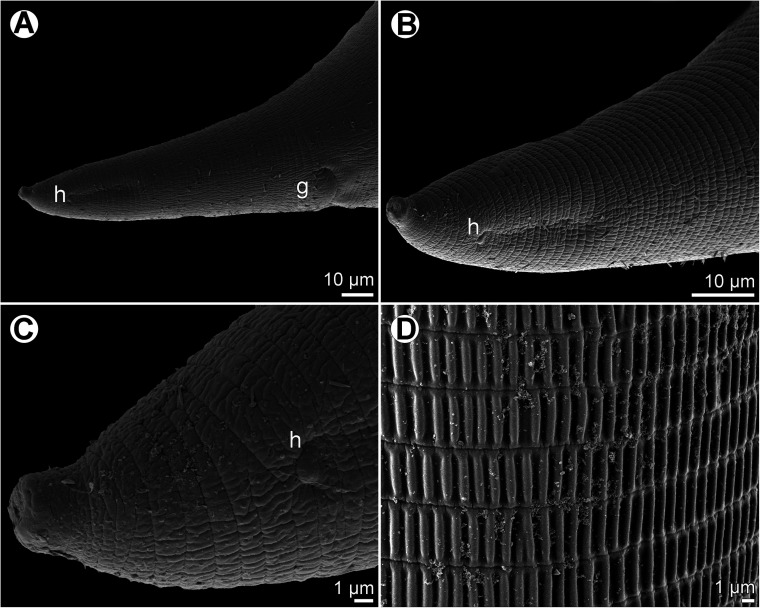
*Ascarophisnema tridentatum* Moravec et Justine, 2010 from *Gymnocranius grandoculis*, scanning electron micrographs of female. (A) Tail, lateral view; (B) posterior part of tail, lateral view; (C) tail tip; (D) detail of cuticle at anterior part of body. (g) Anus; (h) phasmid.

*Female* (1 ovigerous specimen): Small, whitish filiform nematode. Body length 35.39 mm, maximum width of body in caudal region 150. Cuticle thick, with distinct transverse striations (Figs. 8E, 8F and 9A–9D). Cephalic end rounded, with two pseudolabia with terminal protrusions three high (Figs. 7F, 8A–8D and 8F). Oral aperture oval, dorsoventrally elongate, slightly narrowed equatorially (Figs. 7D and 8A–8C). Lateral pseudolabia small, each provided with conspicuous conical terminal protrusion; in apical view, inner parts of pseudolabia only moderately cover mouth; expanded dorsoventrally, each forming 2 (1 dorsolateral and 1 ventrolateral) extensions with distally rounded ends (Figs. 7D, 8A and 8B); in sublateral view, both, somewhat depressed extensions of each pseudolabium appear as tooth-like formations protruding anteriorly from common base of pseudolabium, which grows out of lateral wall of buccal cavity (Figs. 7F and 8A–8D). Submedian sublabia well developed, fused together dorsally and ventrally, forming thus continuous, dorsal and ventral U-shaped inner margins of oral aperture (Figs. 7D, 7F and 8A–8C). Four elongate submedian cephalic papillae and pair of smaller lateral amphids present (Figs. 7D and 8A–8C). Entire vestibule (stoma) including prostom 144 long; well-developed funnel-shaped anterior prostom visible in lateral view (Figs. 7A–7C) 18 long, 15 wide. Length of muscular oesophagus 270, maximum width 27; length of glandular oesophagus 2.22 mm, maximum width 63; length ratio of muscular and glandular parts of oesophagus 1:8. Length of entire oesophagus and vestibule represents 7% of total body length. Nerve ring encircles muscular oesophagus near its anterior end, 195 from anterior extremity; excretory pore located somewhat posterior to level of nerve ring, 273 from anterior end of body; deirids small, trident-like, situated somewhat anterior to mid-length of vestibule length, at 63 from anterior extremity (Figs. 7B, 7E, 8E and 8F). Tail conical, 126 long, with rounded tip bearing small terminal cuticular knob 3 long and 6 wide (Figs. 7H, 7I and 9A–9C); pair of small lateral phasmids present near tail tip (Figs. 7H, 7I and 9A–9C). Vulva postequatorial, situated 21.18 mm from anterior end of body, at 60% of body length; vulval lips not elevated. Vagina short, directed anteriorly from vulva. Anterior ovary and uterus not extending anteriorly to oesophageal part of body; posterior ovary forms coils in region anterior to rectum (Figs. 7A and 7H). Uterus filled with many eggs. Mature eggs (containing larvae) oval, thick-walled, size 39–45 × 24–30; thickness of egg wall 4–5; surface of eggs without filaments (Fig. 7G).

#### Remarks

Since the morphology and measurements of the only available female specimen, including the fine structure of the mouth and the shape of deirids, are much the same as those of *Ascarophisnema tridentatum* (except for a slightly longer body length) described from the congeneric host *Gymnocranius euanus* (Günther) from off New Caledonia [35], this specimen is considered to belong to this species. The present finding of *A*. *tridentatum* in *G*. *grandoculis* represents a new host record.

Yamaguti [61] described *Rhabdochona gymnocranii* Yamaguti, 1935 from two gravid females collected in the stomach of *Gymnocranius griseus* (Temminck et Schlegel) from the Inland Sea, Japan and later the species was transferred to *Ascarophis* van Beneden, 1871 [6]. The species has not been recorded since its original description. Yamaguti’s type specimens were re-examined and illustrated by Moravec [24] and Ko [19], but because these were mounted as permanent slides and some taxonomically important features, in particular detailed cephalic structures, could not be studied on them, this species has been considered to be a *species inquirenda* [35]. Taking into account that *Ascarophis* sp. of Mamaev, 1970, described from *G*. *griseus* of the Gulf of Tonkin near Vietnam [22] which is probably identical with *A*. *gymnocranii* [35], is similar to *Ascarophisnema tridentatum*, the species *Ascarophis gymnocranii* was provisionally transferred to *Ascarophisnema* Moravec et Justine, 2010 [35]. It may well be that future studies on *A*. *gymnocranii* show its identity with *A*. *tridentatum*.
